# The Impact of Reminiscence on Autobiographical Memory, Cognition and Psychological Well-Being in Healthy Older Adults

**DOI:** 10.5964/ejop.v16i2.2097

**Published:** 2020-05-29

**Authors:** Andrew P. Allen, Caoilainn Doyle, Richard A. P. Roche

**Affiliations:** a Trinity College Dublin, Dublin, Ireland; b Department of Psychology, Maynooth University, Maynooth, Ireland; Department of Psychology and Counselling, Webster University Geneva, Geneva, Switzerland

**Keywords:** memory, reminiscence, autobiographical memory, episodic memory, ageing

## Abstract

Reminiscence therapy has improved autobiographical memory in older adults with memory impairment. However, there has been a relative lack of research examining the impact of reminiscence interventions on healthy older adults, despite the fact that healthy ageing has been associated with a reduction in episodic autobiographical memory. The current study examined the effects of a semi-structured reminiscence program, compared to a no-intervention control and an active control group focused on current life, in healthy older adults. Before and after reminiscence or control, we assessed episodic and semantic autobiographical memory, as well as reliving of the memory and re-experiencing the emotion associated with the memory. We also examined new learning and executive function, as well as quality of life, satisfaction with life, anxiety, depression, and mood. The reminiscence intervention did not lead to a differing impact on autobiographical memory, cognition or psychological well-being, compared to the control groups. The current results indicate that simple reminiscence does not lead to enhanced autobiographical memory performance in healthy older adults.

Healthy ageing is a key aim of public health policy. For ageing adults, loss of memory function in general is a source of anxiety; this is a likely explanation for the finding that memory lapses in vignettes tended to be attributed more to greater mental difficulty, if the vignette’s protagonist was an older person rather than a younger person (Erber, Szuchman, & Rothberg, 1990). Despite broad evidence that cognitive training and mental stimulation can benefit cognition in healthy older adults (Kelly et al., 2014), there has been a relative paucity of research on enhancing long-term memory, including autobiographical memories from across the lifespan. Autobiographical memory interacts closely with our sense of self (e.g. Conway & Pleydell-Pearce, 2000; Prebble, Addis, & Tippett, 2013), and reminiscence about autobiographical memory may serve a number of functions in healthy ageing, such as building intimacy with others or communicating advice (Cappeliez, O’Rourke, & Chaudhury, 2005). There is thus need for more research on the impact of reminiscence on autobiographical memory in healthy older adults.

Cross-sectional evidence indicates that *episodic* aspects of autobiographical memory (i.e. those relating to specific occurrences located in a specific place and time) may be more diminished with ageing (or less preferred in verbal reports of memory) compared to *semantic* aspects (i.e. those aspects of memory relating to general knowledge of one’s autobiography; Levine, Svoboda, Hay, Winocur, & Moscovitch, 2002; St. Jacques & Levine, 2007). This suggests there may be specific effects of ageing on different components of autobiographical memory.

Previous research involving people with memory impairment has indicated that reminiscence can improve autobiographical memory (e.g. Lopes, Afonso, & Ribeiro, 2016; Meléndez, Torres, Redondo, Mayordomo, & Sales 2017). Another study did not find an overall effect, although higher engagement with reminiscence was associated with improved autobiographical memory (Woods et al., 2016). There is also evidence that reminiscence can improve mood and depressive symptoms in older adults living in long-term residential care (Wang, 2005), and we have previously suggested that positive effects of reminiscence on autobiographical memory might be mediated by effects on mood and executive function (Allen, Doyle, Commins, & Roche, 2018).

Despite this promising research in people with memory impairment, there has been a relative lack of research examining the effects of reminiscence on autobiographical memory in healthy older adults with a lack of cognitive impairment. Memory can decline even in healthy ageing, albeit with differing effects on differing forms of memory (e.g. Hedden & Gabrieli, 2004). It is thus of interest whether reminiscence can have beneficial effects on cognition and psychological well-being, particularly autobiographical memory. One study that did examine reminiscence and autobiographical memory in healthy older adults tested the effects of both oral reminiscence and autobiographical writing (de Medeiros, Mosby, Hanley, Pedraza, & Brandt, 2011); their results suggested a lack of change in autobiographical memory performance.

In the current study, we evaluated the effect of a reminiscence intervention on autobiographical memory, new learning and executive function, as well as self-reported memory and psychological well-being. In contrast to de Medeiros et al., we assessed memory for a wider range of life epochs; de Medeiros et al. employed three (childhood, early adult life, and recent life), whereas we examined five (0-15, 15-30, 31-45 years of age, 46 years of age-last 5 years, last 5 years). The period from 15-30 years in particular approximately aligns with what is referred to as the *reminiscence bump*, a period in life from adolescence to early adulthood that tends to be well-remembered compared to other periods of life by middle-aged and older adults (e.g. Conway & Rubin, 1993; Jansari & Parkin, 1996). Furthermore, instead of two different reminiscence conditions, we employed an active control that was more focused on the present/future than the past, in order to control for factors such as social contact within a group setting and level of attention from the project staff. We also included a waiting list control to quantify the effects of repeated testing with no intervention. For autobiographical memory, in addition to semantic and episodic memory performance, we assessed autonoetic aspects of autobiographical memories. These are subjective mental “time travel” aspects of memory (e.g. Wheeler, Stuss, & Tulving, 1997), which have been demonstrated to differ between younger and older participants (Piolino et al., 2006) and to differ within older adults depending on time of encoding (Luchetti & Sutin, 2018). To assess autonoetic aspects of autobiographical memory, we specifically assessed self-reported extent of reliving the memory and re-experiencing the emotion associated with the memory.

Our key hypothesis was that reminiscence would be associated with heightened episodic autobiographical memory performance, as well as increased re-experiencing of autobiographical memory, compared to the active control and waiting list control. We also hypothesized that reminiscence would be associated with improved cognitive performance (better performance on tests of executive function and verbal learning) and self-reported psychological well-being (enhanced quality of life, satisfaction with life, positive affect, and reduced negative affect, anxiety, and depression).

## Method

This research received ethical approval from the Maynooth University research ethics committees. All participants provided informed consent before beginning their participation in the project. The methods and primary outcome measures of this study were pre-registered at Open Science Framework (see Supplementary Materials).

### Design

This study employed a mixed factorial design, with reminiscence condition as the between-participants factor and pre- and post-assessment as the within-participants factor. Participants were randomly assigned on a per-site basis to one of three conditions (reminiscence therapy, active control group, waiting list control group). Those in the control groups were offered participation in a reminiscence group following their completion of the post-control research visit.

### Materials

Autobiographical memory was assessed using the Episodic Autobiographical Memory Interview (EAMI; Irish, Lawlor, O’Mara, & Coen, 2008, 2010, 2011). This interview assesses episodic and semantic autobiographical memory, as well as autonoetic aspects of autobiographical memory, for five different life epochs (0-15, 15-30, 31-45 years of age, 46 years of age-last 5 years, last 5 years). Participants were requested to think of different memories than what they had discussed at baseline, or during any reminiscence sessions, when they completed the EAMI for a second time, following the intervention/waiting list. We requested this in order to avoid participants building on any rehearsal of memories they had recently retrieved.

New verbal learning was assessed with the Rey Auditory Verbal Learning Test (RAVLT; Schmidt, 1996). This assesses learning of a 15-word list; there are five trials in which the list is read and participants are required to recall as many words as they can in any order. These five trials are followed by one trial of an interference list. Participants are then requested to recall as many words from the first list, both immediately after the interference trial and at delay (following completion of the EAMI).

We also assessed two aspects of executive function; response inhibition and verbal fluency. We assessed response inhibition using a computerised colour-naming Stroop task (Balota et al., 2010; Doyle, Smeaton, Roche, & Boran, 2018), presented using E-prime^®^. Participants were presented with a series of words and asked to name the colour of ink in which they were presented. Although previous research also included a word-naming block, we omitted this to reduce participant burden. We assessed verbal fluency from the Montreal Cognitive Assessment (MoCA; Nasreddine et al., 2005); participants were required to think of as many words beginning with “F” as they could (or “A” for the parallel version) in one minute.

We employed a number of self-report measures of subjective quality of life and satisfaction with life, mental health and affect; these were the Control, Autonomy, Self-realisation and Pleasure quality of life questionnaire (CASP-19; Netuveli, Wiggins, Hildon, Montgomery, & Blane, 2006); Satisfaction With Life Scale (SWLS; Diener, Emmons, Larsen, & Griffin 1985), Beck Depression Inventory (BDI; Beck, Steer, & Brown, 1996), Spielberger Trait Anxiety Inventory (STAI; Spielberger, Gorsuch, Lushene, Vagg, & Jacobs, 1983), and Positive and Negative Affect Schedule (PANAS; Watson, Clark, & Tellegen, 1988).

### Participants

**Figure 1:**
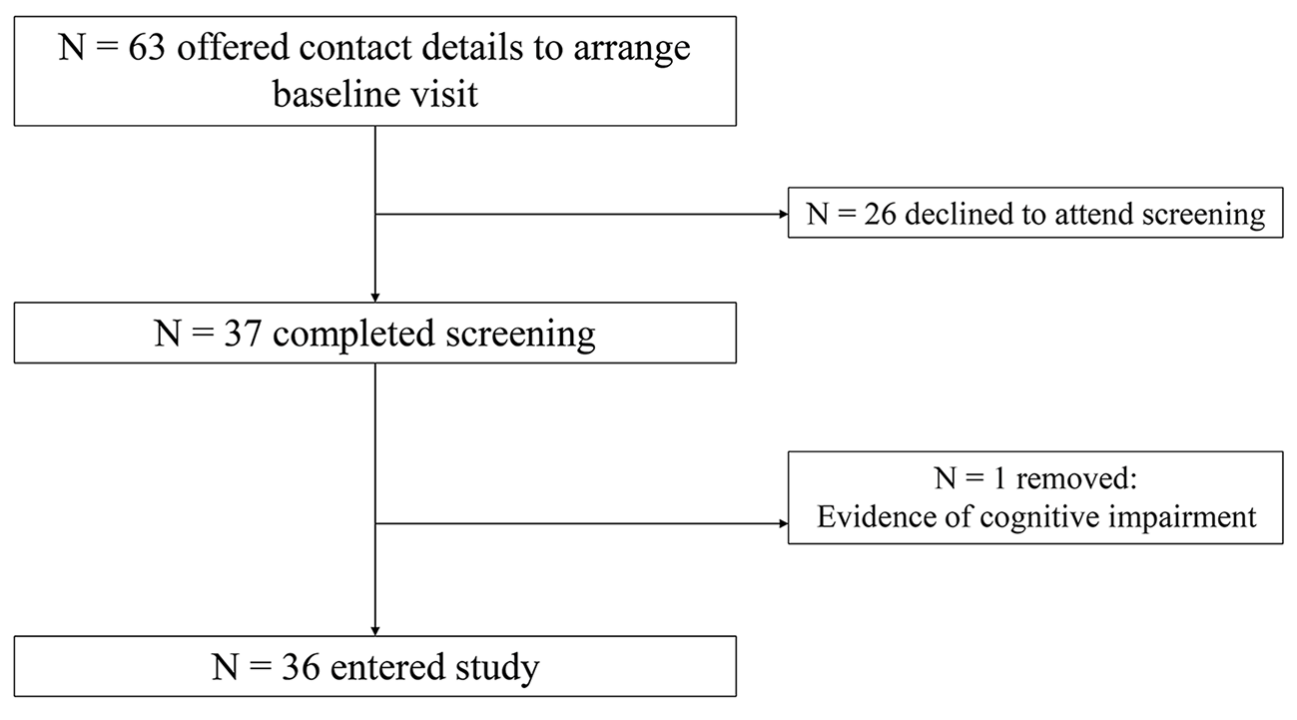
Recruitment flowchart.

We included 36 participants in the analysis for this study; one participant was excluded following baseline assessment due to evidence of cognitive impairment. We assigned 14 to the reminiscence condition (10 females, 4 males; *M*_age_ = 69, *SEM* = 1.3), 11 to the active control (10 female, 1 male, *M*_age_ = 68.8, *SEM* = 1.5) and 11 to the no-intervention control (8 female, 3 male, *M*_age_ = 71.9, *SEM* = 2.0). All participants identified as White Irish. Participants were recruited from Dublin and surrounding counties in the Republic of Ireland, via flyers and newspaper advertisements, as well as via community groups (e.g. active ageing groups, men’s sheds). All participants were over 60 years of age. Exclusion criteria were as follows: severe visual impairments; history of psychological/neurological impairment, severe head trauma resulting in loss of consciousness, history of epilepsy, currently taking psychoactive medication, other relevant medical conditions, history of drug or alcohol problems (See Figure 1 for recruitment flowchart).

### Interventions

The reminiscence and active control interventions were delivered in small groups of three to seven participants at a number of different venues. The reminiscence and active control groups comprised six weekly sessions, each lasting one hour. The reminiscence groups used a simple reminiscence approach, adapted from the “remembering yesterday, caring today” program (Schweitzer & Bruce, 2008). This program was used in previous research examining autobiographical memory as an outcome (Woods et al., 2016). The sessions were more conversation-based than the “remembering yesterday, caring today”, which had a stronger focus on hands-on activities for working with clients with memory problems. We did not target a specific function of reminiscence as we were interested in observing which functions of reminiscence would emerge in the group context.

For the reminiscence groups, in addition to autobiographical memories, participants were prompted to discuss memories of historical events in the public sphere (e.g. John F. Kennedy’s visit to Ireland in the 1960’s, economic recession in Ireland in the 1980’s). For the active control groups, the topics of discussion were approximately matched to the content of the reminiscence groups, but with a focus on the present and future. For example, the active control group discussed learning new things whilst the reminiscence group discussed schooldays. As certain topics were more likely to provoke discussion of different epochs in autobiographical memory, the order of topics for the groups were reversed for half the groups (i.e. half of the reminiscence groups completed the sessions in one order, and the other half of the reminiscence groups completed them in the opposite order). The order of topics was similarly reversed for half of the active control groups. The weekly topics for the reminiscence group and active control group are outlined in Table 1 below.

**Table 1:** Session Topics for Reminiscence Group and Active Control

Session	Reminiscence group	Active control
Session 1	Introduction, childhood and family life	Introduction, health and well-being
Session 2	Schooldays	Continued education/learning new skills
Session 3	Historical session A	Current affairs (national)
Session 4	Homes, gardens and animals, going out and having fun	Holidays and celebrations
Session 5	Historical session B	Current affairs (international)
Session 6	Weddings, babies, children, rounding up	Family life, grandchildren, relationships, rounding up

Reminiscence sessions were recorded, and, with participants’ permission, highlights from the reminiscence groups were added to an online archive at the Digital Repository of Ireland (see Supplementary Materials). Participants were encouraged to listen to previous entries in the archive, to help trigger memories of their own.

### Procedure

Participants completed the cognitive measures in a small testing room one at a time. This testing session lasted approximately 60-90 minutes. Participants completed the questionnaires in their own time prior to the first group session. After completing the reminiscence/active control group, or waiting an equivalent amount of time in the waiting list condition, participants completed the same measures again, 6-8 weeks after the baseline testing session. Parallel versions of tests were used wherever available for the post-intervention study visit, to minimise learning or other carryover effects.

### Statistical Analysis

Where assumptions of parametric data were violated, we transformed variables using a square root transformation; where that transformation did not make the data parametric then a natural log transformation was used. Data for participants who competed the baseline assessment but were lost to follow-up were imputed using last observation carried forward.

We used ANCOVA for the primary outcome (autobiographical memory) and MANCOVA (using Roy’s largest root; see Field, 2009) for the secondary outcomes, which were executive function (verbal fluency and Stroop effect reaction time), verbal learning (RAVLT performance indices) and psychological well-being (quality of life, satisfaction with life, depression, anxiety, positive and negative affect). We employed reminiscence condition and pre-post assessment as predictors and age as a covariate. We assessed the interaction between the two predictors to determine if the change in outcome from baseline to post-intervention differed between the reminiscence group and the control conditions. We also examined whether there was a main effect of life epoch on autobiographical memory.

## Results

### Autobiographical Memory

**Figure 2:**
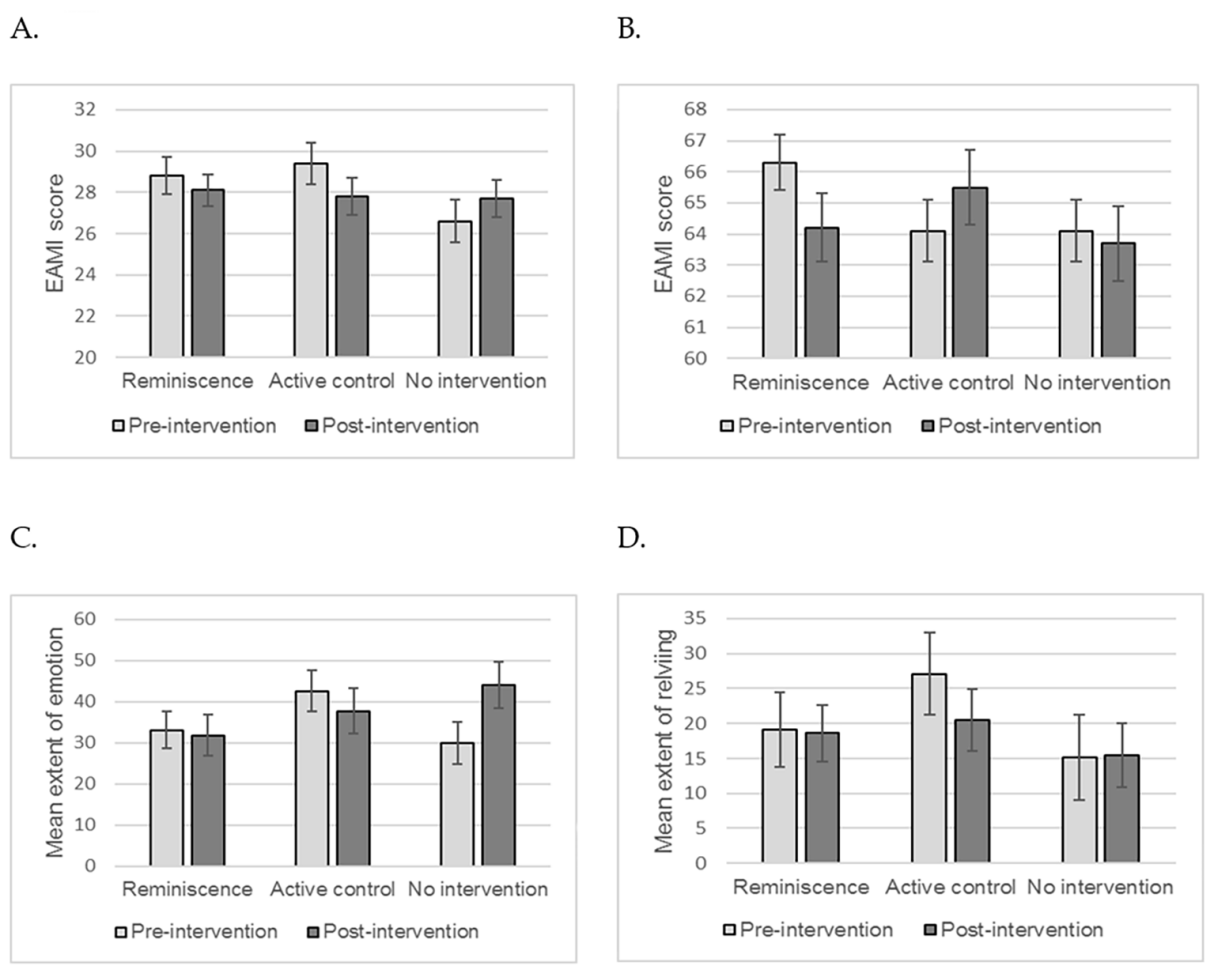
Autobiographical memory pre- and post-intervention condition. *Note*. (A) - episodic memory, (B) - semantic memory, (C) - re-experiencing emotion, (D) - reliving the memory. Estimated marginal means adjusted for covariate (age) are reported. Error bars represent standard error of the mean.

Autobiographical memory was the primary outcome assessed within this study. There was a significant interaction between pre-post assessment and reminiscence condition for episodic memory, *F*(2, 32) = 3.98, *p* = .03, $\eta_{p}^{2}$ = .2; this was due to a fall in episodic memory retrieval in the active control group, rather than a change in the reminiscence group. There was no significant main effect of reminiscence condition, *F*(2, 32) = 0.72, *p* = .49, $\eta_{p}^{2}$ = .04. However, there was a main effect of pre-post assessment, *F*(1, 32) = 6.46, *p* = .02, $\eta_{p}^{2}$ = .17, with participants performing slightly worse at post-intervention regardless of reminiscence condition (see Figure 2A).

There was no significant effect of reminiscence condition on semantic autobiographical memory; there no significant interaction between pre-post assessment and reminiscence condition, *F*(2, 32) = 2.67, *p* = .09, $\eta_{p}^{2}$ = .14, nor was there a main effect of reminiscence condition, *F*(2, 32) = 0.54, *p* = .59, $\eta_{p}^{2}$ = .03 or pre-post assessment, *F*(1, 32) = 0.08, *p* = .78, $\eta_{p}^{2}$ = .002 (see Figure 2B).

Despite an increase in the no intervention condition, there was no significant interaction between pre-post assessment and reminiscence condition for extent of re-experiencing emotion associated with autobiographical memory, *F*(2, 32) = 2.49, *p* = .1, $\eta_{p}^{2}$ = .14. There was a marginally significant main effect of pre-post assessment, *F*(1, 32) = 4.16, *p* = .05, $\eta_{p}^{2}$ = .12, with participants generally reporting higher levels of re-experiencing emotion at post-intervention. There was no significant main effect of reminiscence condition on re-experiencing emotion, *F*(2, 32) = 0.94, *p* = .4, $\eta_{p}^{2}$ = .06 (see Figure 2C).

There was no significant interaction between pre-post assessment and reminiscence condition for extent of reliving the memory, *F*(2, 32) = 0.2, *p* = .82, $\eta_{p}^{2}$ = .01. There was a marginally significant main effect of pre-post assessment, *F*(1, 32) = 3.89, *p* = .06, $\eta_{p}^{2}$ = .11, with participants generally reporting lower levels of reliving the memory at post-intervention. There was no significant main effect of reminiscence condition on reliving the memory, *F*(2, 32) = 1.03, *p* = .37, $\eta_{p}^{2}$ = .06 (see Figure 2D).

**Figure 3:**
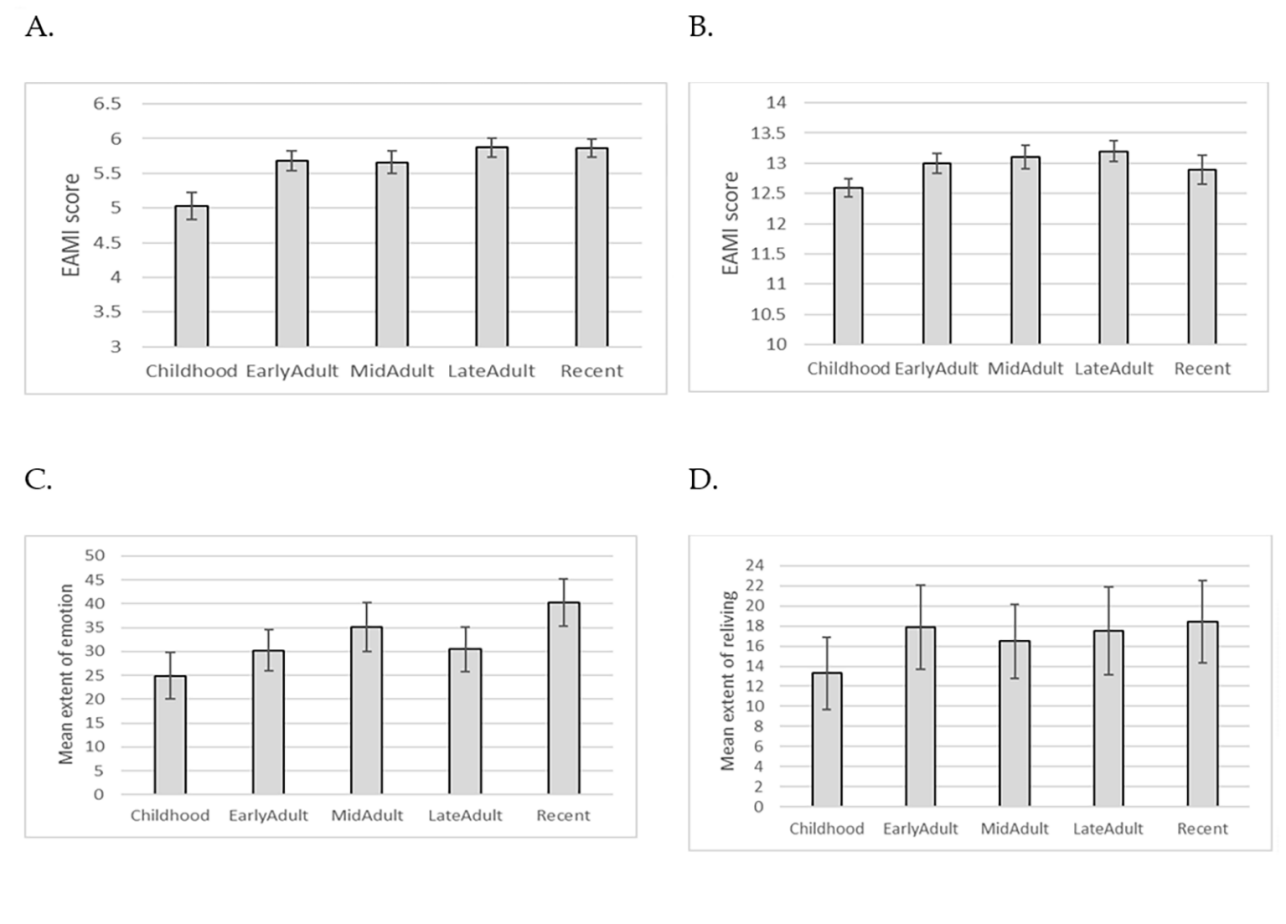
Autobiographical memory across epoch for (A) - episodic memory, (B) - semantic memory, (C) - re-experiencing emotion, (D) - reliving the memory. *Note.* Estimated marginal means adjusted for covariate (age) are reported. Error bars represent standard error of the mean.

There was some evidence of lower autobiographical memory performance for the childhood period (0-15 years), although there was no significant main effect of life epoch on episodic memory, *F*(4, 128) = 0.69, *p* = .60, $\eta_{p}^{2}$ = .02 (see Figure 3A), or semantic memory, *F*(3.05, 97.52) = 0.67, *p* = .57, $\eta_{p}^{2}$ = .02 (Greenhouse-Geisser adjusted; see Figure 3B).

Life epoch did have a main effect on re-experiencing of emotion, *F*(4, 104) = 3.14, *p* = .02, $\eta_{p}^{2}$ = .11, with participants reporting less re-experiencing of emotion for childhood memories (see Figure 3C). Despite a simliar trend for reliving of the event, there was not a significant main effect of life epoch, *F*(4, 104) = 2.2, *p* = .07, $\eta_{p}^{2}$ = .08 (see Figure 3D). These main effects were not significantly moderated by condition or pre-post assessment.

### Cognition

Using Roy’s Largest Root, there was no significant interaction between pre-post assessment and reminiscence condition for executive function (verbal fluency and Stroop effect RT), Θ = .12, *F*(2, 28) = 1.72, *p* = .2, $\eta_{p}^{2}$ = .11. There was no significant main effect of pre-post assessment Θ = .03, *F*(2, 27) = 0.35, *p* = .71, $\eta_{p}^{2}$ = .03, nor was there a significant main effect of reminiscence condition, Θ = .17, *F*(2, 28) = 2.32, *p* = .12, $\eta_{p}^{2}$ = .14 (see Table 2).

**Table 2:** Executive Function Before and After Reminiscence Intervention and Control Conditions

Outcome / Condition	Pre	Post
Verbal fluency (words produced)		
Reminiscence	16.28 (1.31)	16.25 (1.19)
Active Control	14.04 (1.34)	12.48 (1.21)
No intervention	14.14 (1.57)	11.74 (1.42)
Stroop effect RT (ms)		
Reminiscence	201.94 (32.46)	192.45 (42.59)
Active Control	178.88 (33.07)	165.62 (43.39)
No intervention	193.97 (38.97)	118.04 (51.12)

*Note*. Estimated marginal means reported; Standard error of the mean in parentheses.

There was no significant interaction between pre-post assessment and reminiscence condition for new learning, Θ = .23, *F*(7, 27) = 0.88, *p* = .53, $\eta_{p}^{2}$ = .19. There was no significant main effect of pre-post assessment, Θ = .31, *F*(7, 26) = 1.15, *p* = .37, $\eta_{p}^{2}$ = .24, nor was there a main effect of reminiscence condition, although this approached significance, Θ = .57, *F*(7, 27) = 2.21, *p* = .07, $\eta_{p}^{2}$ = .36. (see Table 3).

**Table 3:** New Verbal Learning Before and After Reminiscence Intervention and Control Conditions

Outcome / Condition	Pre	Post
RAVLT trial 1 correct		
Reminiscence	6.18 (0.41)	6.73 (0.36)
Active Control	5.75 (0.46)	5.87 (0.4)
No intervention	6.4 (0.47)	5.8 (0.41)
RAVLT trial 5 correct		
Reminiscence	11.64 (0.64)	11.22 (0.57)
Active Control	10.82 (0.71)	10.82 (0.63)
No intervention	10.82 (0.73)	10.26 (0.65)
RAVLT trial 6 correct		
Reminiscence	9.78 (0.73)	9.04 (0.8)
Active Control	8.22 (0.82)	7.71 (0.89)
No intervention	8.06 (0.84)	7.7 (0.91)
RAVLT delayed recall correct		
Reminiscence	10.03 (0.85)	8.38 (0.87)
Active Control	8.66 (0.95)	7.52 (0.97)
No intervention	8.39 (0.97)	7.18 (1)
RAVLT learning		
Reminiscence	5.47 (0.74)	4.49 (0.62)
Active Control	5.07 (0.83)	4.95 (0.69)
No intervention	4.42 (0.86)	4.42 (0.71)
RAVLT forgetting		
Reminiscence	0.13 (0.06)	0.25 (0.06)
Active Control	0.2 (0.06)	0.31 (0.07)
No intervention	0.25 (0.06)	0.34 (0.07)
RAVLT interference		
Reminiscence	0.16 (0.05)	0.19 (0.06)
Active Control	0.24 (0.06)	0.29 (0.07)
No intervention	0.26 (0.06)	0.28 (0.07)
RAVLT total repeats		
Reminiscence	4.4 (0.98)	3.16 (0.7)
Active Control	2.98 (1.09)	4.33 (0.8)
No intervention	3.15 (1.12)	3.56 (0.82)

*Note*. Estimated marginal means reported; Standard error of the mean in parentheses.

### Psychological Well-Being

There was no significant interaction between pre-post assessment and reminiscence condition for psychological well-being, Θ = .14, *F*(6, 14) = 0.33, *p* = .91, $\eta_{p}^{2}$ = .12. There was no significant main effect of pre-post assessment, Θ = .92, *F*(6, 13) = 1.99, *p* = .14, $\eta_{p}^{2}$ = .48, nor was there a main effect of reminiscence condition, although this approached significance, Θ = 1.2, *F*(6, 14) = 2.8, *p* = .053, $\eta_{p}^{2}$ = .55 (see Table 4).

**Table 4:** Psychological Well-Being Before and After Reminiscence Intervention and Control Conditions

Outcome / Condition	Pre	Post
Positive affect		
Reminiscence	37.7 (2.12)	40.71 (2.29)
Active Control	35.5 (2.51)	40.24 (2.7)
No intervention	37.8 (2.19)	39.6 (2.36)
Negative affect		
Reminiscence	14.76 (1.77)	14.11 (1.25)
Active Control	14.19 (2.1)	13.61 (1.48)
No intervention	15.98 (1.84)	17.55 (1.29)
Depression		
Reminiscence	5.93 (1.84)	5.04 (1.86)
Active Control	7.52 (2.17)	7.65 (2.2)
No intervention	7.56 (1.9)	7.47 (1.92)
Anxiety		
Reminiscence	33.13 (2.83)	29.83 (2.65)
Active Control	30.46 (3.35)	29.93 (3.13)
No intervention	33.41 (2.93)	31.59 (2.74)
Quality of life		
Reminiscence	41.81 (1.02)	40.54 (1.09)
Active Control	39 (1.21)	37.91 (1.29)
No intervention	41.43 (1.06)	40.74 (1.13)
Satisfaction with life		
Reminiscence	28.07 (1.1)	29.06 (1.51)
Active Control	26.48 (1.3)	27.27 (1.79)
No intervention	25.45 (1.14)	25.64 (1.56)

*Note*. Estimated marginal means reported; Standard error of the mean in parentheses.

## Discussion

The current findings do not suggest that reminiscence affects autobiographical memory in healthy older adults, compared to an active control and no intervention control. This is consistent with previous research contrasting oral and written reminiscence (de Medeiros et al., 2011). There was also a lack of evidence that reminiscence was associated with better performance on other cognitive tasks, or with improved psychological well-being. Cognitive performance and psychological well-being were generally good at baseline, and, in the case of semantic autobiographical memory, quite close to ceiling, consistent with previous evidence that semantic autobiographical memory is well preserved in healthy ageing (Levine et al., 2002; St. Jacques & Levine, 2007). Thus, although the current study had a low sample size, given the high level of performance at baseline it is unlikely that improvement would be demonstrated with a larger sample. The slight decline observed for some autobiographical memory measures may be due to participants being requested to think of a different memory following the intervention; they may have selected an autobiographical memory that had been less well-rehearsed prior to the project, or was less vivid than the memory they discussed at baseline.

Future research may determine if there are other means through which interventions may better help to increase autobiographical memory in older adults. A greater use of multimedia resources, or integrating reminiscence with other memory interventions (e.g. those usually more focused on short-term memory and learning) may lead to an enhanced effect on autobiographical memory. Ongoing intervention with long-term follow-up may help to determine if there are preventative effects of reminiscence in healthy older adults at risk of mild cognitive impairment or dementia. Although we employed simple reminiscence, more targeted reminiscence interventions may have more specific effects; for example, instrumental reminiscence focused on past experiences of coping has been shown to have a positive effect on resilience and coping strategies (Meléndez, Fortuna, Sales, & Mayordomo, 2015). However, we did not attempt to focus on a specific function of reminiscence in the current project, as we were interested in observing which functions of reminiscence would emerge in the group context. Given that there are different functions of reminiscence (e.g. Cappeliez et al., 2005), it would be of interest to see how older adults use discursive practises in reminiscing to acheive different aims.

In conclusion, the current results are consistent with previous findings that a reminiscence intervention does not lead to enhanced autobiographical memory performance in healthy older adults (de Medeiros et al., 2011). Further research is required to ascertain the best means by which to preserve episodic memory in healthy ageing. We should also note that, even in the absence of individual psychological effects, participating in group reminiscence allows for healthy older adults to engage in an activity which may benefit themselves and those around them in other ways, such as preservation of oral history (e.g. Bornat, 1989).

## Data Availability

Data for this article is freely available (see the Supplementary Materials  section).

## Notes

The following Supplementary Materials are available:

1. The methods and primary outcome measures of this study were pre-registered at Open Science Framework and are available via https://osf.io/9zd5u/.
2. Reminiscence sessions were recorded, and, with participants’ permission, highlights from the reminiscence groups were added to an online archive at the Digital Repository of Ireland and are available via https://repository.dri.ie/catalog/wp98p078n. Participants were encouraged to listen to previous entries in the archive, to help trigger memories of their own.
